# Supplementary material for: Tamoxifen therapy in a murine model of myotubular myopathy
Source: Nat Commun. 2018 Nov 19;9:4849. doi: 10.1038/s41467-018-07057-5 (PMC6242823; doi:10.1038/s41467-018-07057-5)

Tamoxifen Therapy in a Murine Model of Myotubular Myopathy

Maani et al.

Supplementary Dataset 1. Spreadsheet of transcripts significantly enriched with comparative RNA sequencing. RNA sequencing was performed on skeletal muscle extracts from wild type (WT) and Mtm1 knockout (KO) mice +/- tamoxifen (TAM) treatment. Studies were performed at 35 days of age on samples from littermates, with treatment or placebo started at 21 days of age. The excel sheet presents all significantly changed transcripts in the following comparisons: WT vs KO, WT vs WT+TAM, KO vs KO+TAM, and WT+TAM vs KO+TAM.

Supplementary Dataset 2. Summary of GO terms from comparative RNA sequencing.
GO terms cluster analysis was performed from the RNA sequencing data. Comparisons are made between WT vs. KO, WT vs. WT+TAM, WT+TAM vs. KO+TAM. Of note, there were no GO terms significantly enriched in the comparison between WT and WT+TAM.

Table 1 **Levels of Tamoxifen and metabolites in plasma and hamstring muscle of high and low dose treated WT and Mtm1 KOs**

	Plasma (ng/mL)				Hamstring (ng/g)			
	High-dose TAM		Low-dose TAM		High-dose TAM		Low-dose TAM	
Compound	WT, n= 6	KO, n=5	WT, n=5	KO, n=7	WT, n=7	KO, n=7	WT, n=5	KO, n=7
(E)-Tamoxifen	10.0 ± 2.1	35.0 ± 20	0.3 + 0.0.3	0.3 + 0.03	129.1 ± 8.0	208.2 ± 30.0	5.4 ± 0.3	9.1 ± 0.7
(Z)-Tamoxifen	10.3 ± 3.5	18.7 ± 9.0	0.2 + 0.03	0.14 + 0.06	296.2 ± 41	302.2 ± 66.0	1.7 ± 0.3	3.0 ± 0.8
(E)- 4-Hydroxytamoxifen	17.0 ± 5.0	14.0 ± 6.0	0.3 + 0.03	0.4 + 0.2	137.3 ± 8.0	240.6 ± 39.0	4.0 ± 0.2	6.3 ± 0.4
(Z)- 4-Hydroxytamoxifen	23.5 ± 12.0	20.4 ± 7.4	0.2 + 0.03	0.21 + 0.1	344.0 ± 78	273.6 ± 47.0	1.0 ± 0.2	1.4 ± 0.6
(E)-N-Desmethyltamoxifen	8.1 ± 2.5	26 ± 16.0	0.3 + 0.06	0.3 + 0.04	71.0 ± 8.0	101.3 ± 21.2	3.0 ± 0.1	4.0 ± 0.3
(Z)-N-Desmethyltamoxifen	9.6 ± 2.0	8.0 ± 2.1	0.2 + 0	no detection	183.0 ± 23.3	131.7 ± 27.0	0.4 ± 0.1	0.4 ± 0.02
(E&Z)-Endoxifen	27 ± 8.0	27.0 ± 6.0	no detection	no detection	191.0 ± 37.0	178.0 ± 19.3	2.3 ± 0.2	3.0 ± 0.2

Values represents mean ± SEM

Supplementary Table 1. Levels of tamoxifen and its metabolites.
Levels of tamoxifen and its metabolites were measured by HPLC from extracts derived from plasma or skeletal muscle. Levels were measured at 35 days, two weeks after therapy initiation. Note the dose dependent increase in levels with low and high dose tamoxifen treatment.

Figure 2 Data Table				
**Figure 2B**	**WT vs. KO**	**WT vs. WT+ high TAM**	**KO vs. KO+ high TAM**	**Low TAM**
	n=3 (38 ±3.5μm,); n=4 (19 ±2.2μm); **** p< 0.0001	n=3 (38 ±3.5μm,); n=4 (29 ±1.1μm); *p=0.0178	n=4 (19 ±2.2μm,); n=6 (28 ±2.0μm); **p=0.0065	WT vs. KO: ****p <0.0001
**Figure 2C**	**WT vs. KO**	**WT + high TAM vs. KO + high TAM**	**KO vs. KO + high TAM**	**Low TAM**
	n=3 (0%), n=4 (3± 0.8%) *** p=0.004	n=4 (0%), n=5 (1.1± 0.09%)	n=4 (3± 0.8%); n=5 (1.1± 0.09%), ** p=0.0024	WT: n=4, (0.17± 0.1%) WT+TAM: n= 3 (0.13± 0.06%) KO: n=4 (2.8± 0.8%) *p=0.0120(compared to WT) KO+TAM: n=4 (1.7± 0.2), ns compared to KO
**Figure 2D**	**WT**	**WT+ high TAM**	**KO**	**KO+ high TAM**
	n=3 (0%)	n=3 (0%)	n=3 (60.3± 13.0%) ***p=0.0002 compared to WT ***p = 0.0005 compared to KO+TAM	n= 3 (9.0 ± 0.6%), ns compared to WT+TAM
**Figure 2D**	**WT**	**WT + low TAM**	**KO**	**KO+ low TAM**
	n=4 (0%)	n=3 (0%)	n=3 (66.3± 11.2%) ****p=<0.0001 compared to WT and ****p<0.0001 compared to KO+TAM	n= 4 (12.3 ± 3.7%), ns compared to WT+TAM

Supplementary Table 2. Data points from the graphical values in Figure 2 (histopathology of MTM KO vs. MTM KO + tamoxifen). Specific values for the data points that compose the dot plots in Figure 2B-D. Figure 2B is average myofiber diameter. Figure 2C is the percentage of central nuclei per 100 fibers. Figure 2D is the quantification of dysferlin subcellular localization. WT = wild type; KO = *Mtm1* knockout; TAM = tamoxifen.

Figure 5 Data Table				
**Figure 5A**	**KO vs. KO+EST**			
	42 days (n=5), 56-59 days (*p=0.03)			
**Figure 5D**	**WT+DMSO**	**WT + EST**	**KO+DMSO**	**KO+EST**
	1.00 +/- 6% (n=5)	93 +/- 7% (n=5)	74 +/- 7% (n=3)	69 +/- 6% (n=5)
**Figure 5D**	**WT+MIG**	**WT + FULV**	**KO+MIG**	**KO+FULV**
	1.00 +/- 7% (n=2)	101 +/- 8% (n=4)	56 +/- 8% (n=3)	75 +/- 9% (n=4)
**Figure 5F**	**WT+DMSO**	**WT+EST**	**KO+DMSO**	**KO+EST**
	37 +/- 1.4μm (n=6)	39 +/- 2.1μm (n=3)	22 +/- 1.6μm (n=5), ****p<0.001 compared to WT+DMSO	23 +/- 3μm (n=4); ns compared to KO+DMSO
**Figure 5F**	**WT + MIG**	**WT+ FULV**	**KO+ MIG**	**KO+ FULV**
	39.5 +/- 0.5μm (n=2)	39 +/- 1.7μm (n=3)	18.3 +/- 1.8μm (n=3)	21 +/- 2μm (n=4)
**Figure 5G**	**WT+DMSO**	**WT+EST**	**KO+DMSO**	**KO+EST**
	100% cell membrane, 0% cytoplasmic (n=4)	100% cell membrane, 0% cytoplasmic (n=2)	41 +/- 30% cell membrane, 59 +/- 21% cytoplasmic (n=3) **p=0.048 compared to WT+DMSO	31 +/- 8% cell membrane, 69 +/- 8% cytoplasmic (n=3)
**Figure 5I**	**WT+DMSO**	**WT+EST**	**KO+DMSO**	**KO+EST**
	19 +/- 1.7 (n=3)	15 +/- 2.5 (n=3)	0.5 +/- 0.4 (n=2); *p=0.0151 compared to WT+ DMSO	7 +/- 6 (n=3)

Supplementary Table 3. Data points from the graphical data in Figure 5 (treatments with estradiol and fulvestrant). Values for graphical data in Figure 5. Figure 5A values are median survival. Figure 5D is grip strength values. Figure 5F is myofiber size. Figure 5G is dysferlin localization quantification. Figure 5I is quantification of electron micrographs (triads per field). EST = estradiol, MIG = miglyol, FULV = fulvestrant. KO = *Mtm1* knockout; WT = wild type.

Figure 6 Data Table				
	**WT**	**WT + high TAM**	**KO**	**KO+ high TAM**
**Figure 6D**	1.00 +/- 0.55 (n=5)	0.75 +/- 0.38 (n=5)	9.80 +/- 1.97 (n=5); ***p=0.0002 compared to WT	4.10 +/- 0.80 (n=5); *p=00112 compared to KO
	**WT**	**WT + low TAM**	**KO**	**KO+ low TAM**
**Figure 6E**	1.00 +/- 0.32 (n=4)	0.93 +/- 0.31 (n=4)	9.45 +/- 2.67 (n=4); **p=0.0049 compared to WT	1.80 +/- 1.24 (n=4); **p=0.0097 compared to KO
	**WT + DMSO**	**WT + EST**	**KO + DMSO**	**KO + EST**
**Figure 6F**	1.00 +/- 0.40 (n=4)	1.05 +/- 0.41 (n=4)	5.71 +/- 1.51 (n=4); *p=0.0114 compared to WT	1.32 +/- 0.66 (n=4); *p=0.0280 compared to KO+DMSO

Supplementary Table 4. Data points for graphical data in Figure 6D-F (estrogen receptor alpha levels). Individual data points for Figure 6. Figure 6D-F are the densitometric values for estrogen receptor alpha western blots (normalized to beta actin), with wild type (WT) values set as 1. KO = *Mtm1* knockout; TAM = tamoxifen; EST = estradiol.

Figure 8 Data Table					
**Figure 8E**	**WT**	**WT+ high TAM**	**KO**	**KO+ high TAM**	
	1.00 +/- 0.18 (n=4)	0.45 +/- 0.0065 (n=4); *p=0.0025 compared to WT	2.21 +/- 0.26 (n=4); **p=0.0025 compared to WT	1.05 +/- 0.17 (n=4); **p=0.0027 compared to KO	
**Figure 8F**	**WT**	**WT+ low TAM**	**KO**	**KO+ low TAM**	
	1.00 +/- 0.14 (n=4)	0.45 +/- 0.097 (n=4); *p=0.025 compared to WT (t-test)	2.74 +/- 0.097 (n=4); ***p<0.0001 compared to WT	0.76 +/- 0.29 (n=4); ***p<0.0001 compared to KO	
**Figure 8G**	**WT+DMSO**	**WT+ EST**	**KO+ DMSO**	**KO + EST**	
	1.00 +/- 0.061 (n=3)	0.72 +/- 0.12 (n=3)	1.85 +/- 0.23 (n=3); **p=0.0028 compared to WT+ DMSO	1.47 +/- 0.16 (n=3); **p=0.0067 compared to KO+ DMSO	
**Figure 8H**	**WT + MIG**	**WT+ FULV**	**KO+ MIG**	**KO+ FULV**	
	1.00 +/- 0.21 (n=3)	0.93 +/- 0.31 (n=3)	2.40 +/- 0.23 (n=3); *p=0.043 compared to WT+ MIG	1.98 +/- 0.46 (n=3); *p=ns	
**Figure 8K**	**Control vs. MTM**	**10uM Control vs. MTM+ 10um TAM**			
	2.29 +/- 0.21 (n=3); *p=0.0170	1.29 +/- 0.33 (n=3); **p=0.0044			
**Figure 8L**	**Control vs. MTM**	**10uM Control vs. MTM+ 10um TAM**			
	1.439 +/- 0.08 (n=3), (n=2); *p=0.0494	0.795 +/- 0.003 (n=3), (n=2); *p=0.00176			
**Figure 8O**	**5uM TAM vs. DMSO**	**10uM vs. DMSO**			
	0.89 +/- 0.028 (n=3); p=ns	0.52 +/- 0.13 (n=3); *p=0.0125			
**Figure 8P**	**10uM TAM vs. DMSO**				
	0.71 +/- 0.016 (n=9); *p=0.017				
**Figure 8S**	**10uM TAM vs. DMSO**				
	0.90 +/- 0.1329 (n=4); p=ns				
**Figure 8T**	**10uM TAM vs. DMSO**	**30nM BZ vs. 10uM TAM**	**10uM MG132 vs. 10uM TAM**	**10uM MG132+ 10uM TAM vs. 10uM TAM**	**30nM BZ + 10uM TAM vs. 10uM TAM**
	0.59 +/- 0.030 (n=13), (n=6); *p=0.026	1.29 +/- 0.11 (n=15), (n=13); ****p<0.0001	1.20 +/- 0.11 (n=7), (n=13); *****p<0.0001	1.02 +/- 0.076 (n=7), (n=13); **p=0.01	1.04 +/- 0.11 (n=11), (n=13); **p=0.001

Supplementary Table 5. Data points from graphical data in Figure 8 (dynamin-2 levels). Values for the densitometric quantification of dynamin-2 (DNM2) protein levels under various conditions. Figure 8E-H represents data from DNM2 measurements in mouse skeletal muscle.

TAM = tamoxifen; EST = estradiol; FULV = fulvestrant; WT = wild type; KO = *Mtm1* knockout. Figure 8K and 8L are data from primary human fibroblasts and transdifferentiated myotubes, respectively (MTM = patient with myotubular myopathy). Figure 8O is from C2C12 cells, 8P is from MCF7 cells, and 8S is from HEK293T cells. Figure 8T is from MCF7 cells +/- tamoxifen and +/- proteasomal inhibitors (bortezomib = BZ).

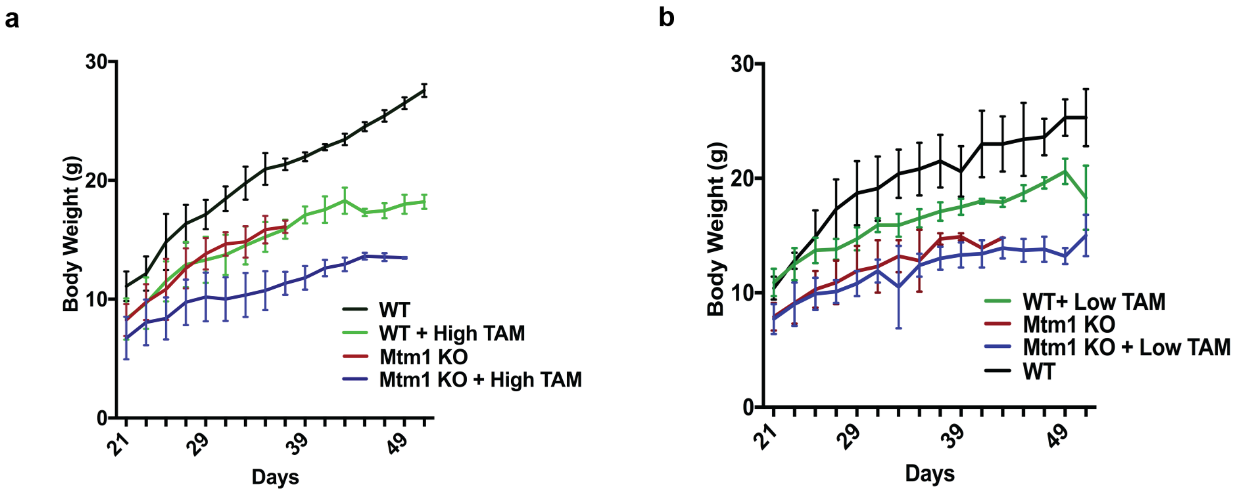

Supplementary Figure 1. Tamoxifen treatment does not improve the body weight of *Mtm1* KO mice. a. Drug treatment with high or low-dose tamoxifen (TAM) does not restore the weight of *Mtm1* KOs. Plots of body weight for high-dose TAM treated animals show that high-dose TAM treated *Mtm1* KOs (n=13) starting at 21 days of age are smaller in weight compared to *Mtm1* KOs alone (n=5), same as high dose TAM treated WTs (n=10) compared to WT alone (n=6). **b.** Plots of body weight for animals treated with low-dose TAM show that low-dose TAM treated *Mtm1* KOs (n=15) are similar in weight compared to *Mtm1* KOs alone (n=6) but lower than low-dose TAM treated WTs (n=9) and WT alone (n=5).

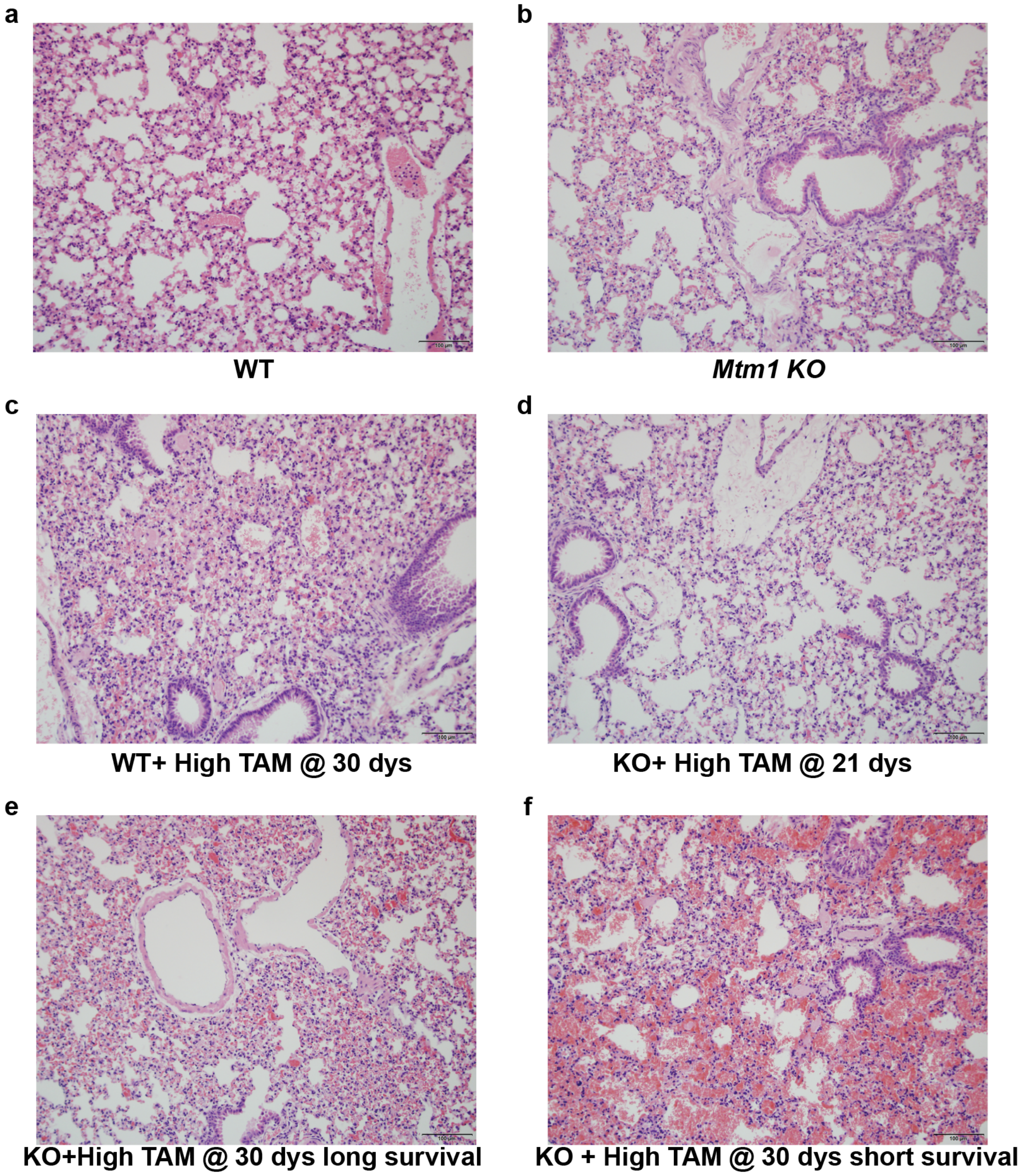

Supplementary Figure 2. Late treatment with high-dose TAM is associated with pulmonary hemorrhage in a subset of *Mtm1* KO animals.

Full necropsy was performed on mice by a veterinary pathologist (n=2 per condition). Depicted are sections from lungs stained with H&E (scale bar, 100 µm). **a.** Untreated wild type littermates sacrificed at 33 days. Mild congestion of the alveolar walls was noted. **b**. End-point untreated *Mtm1* KO with mild congestion of alveolar walls. **c.** Wild type treated with high-dose TAM starting at 30 days and sacrificed at 33 days. Mild congestion of the alveolar walls was observed. **d**. *Mtm1* KO treated with high-dose TAM starting 21 days and terminated at 29 days. Mild congestion of alveolar walls similar to untreated KO mice was observed. **e.** End-point *Mtm1* KO (age 40 days) treated with high-dose TAM starting 30 days (long survival). Pathology shows moderate congestion of the alveolar walls. **f.** *Mtm1* KO treated with high-dose TAM starting 30 days and with precipitant death after 3 days of treatment. Pathology shows severe lung damage and the presence of pulmonary hemorrhage. Scale bars = 100µM

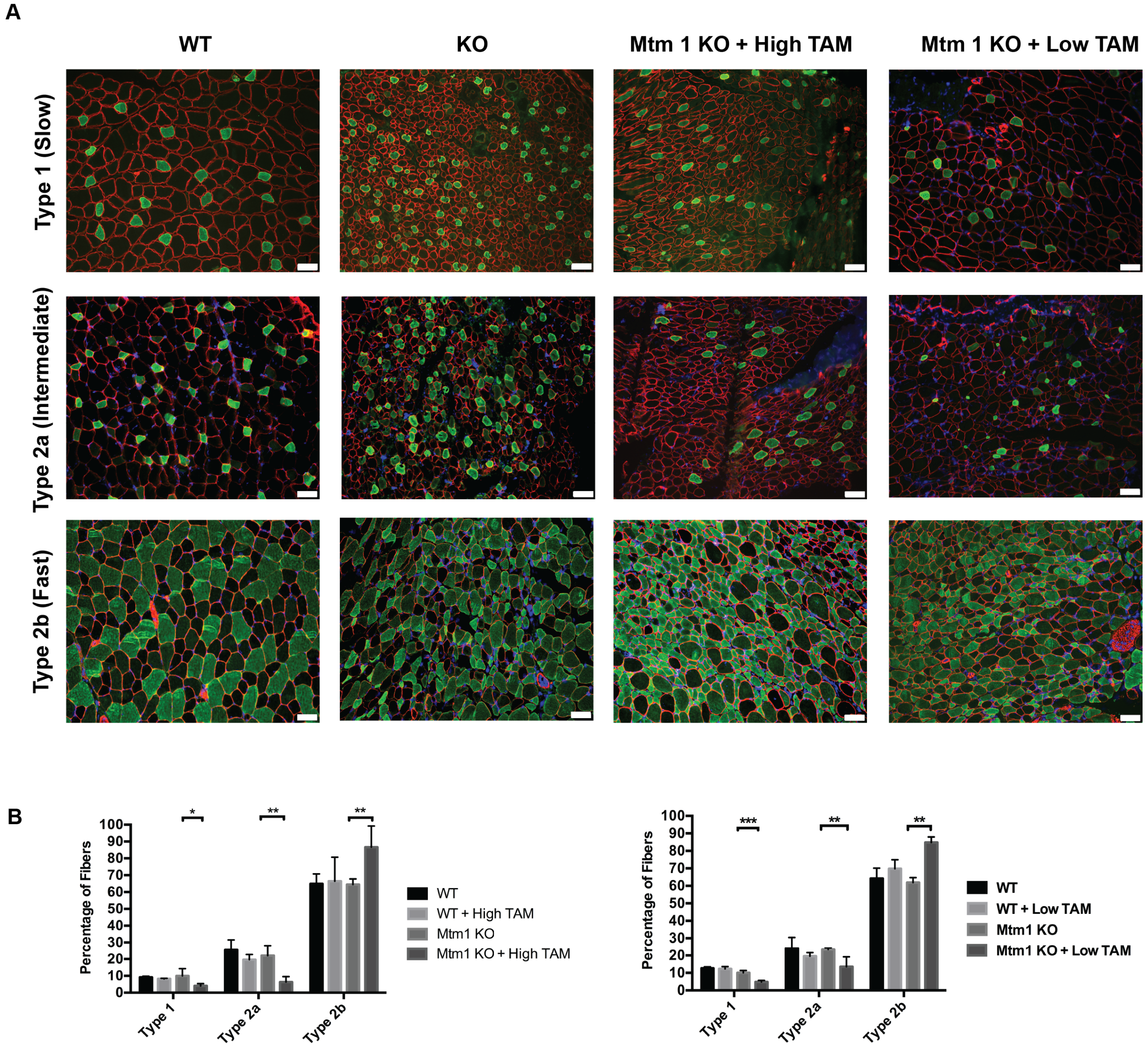

Supplementary Figure 3. High and low-dose tamoxifen treatment result in muscle fiber-type switching. a. Immunofluorescence staining of cross sections from tibialis anterior muscle tissue at 36 days of age for dystrophin (red), myosin heavy chain type 1 (slow), type 2a (intermediate), or type 2b (fast, green) and DAPI (blue), scale bar = 50 µm. **b.** Percentage of fiber type 1, 2a, and 2b in WT and *Mtm1* KO mice treated with both high and low-dose TAM, respectively. *Mtm1* KO mice treated with high-dose TAM display a decrease in type 1 and 2a fibers, and an increase in type 2b fibers (n=6) compared to *Mtm1* KOs (n=3), ** P <0.01. Likewise, *Mtm1* KO mice treated with low-dose TAM also display a decrease in type 1 and 2a fibers, and an increase in type 2b fibers (n=4) compared to *Mtm1* KOs (n=3), ** P <0.01. Scale bars = 20µM

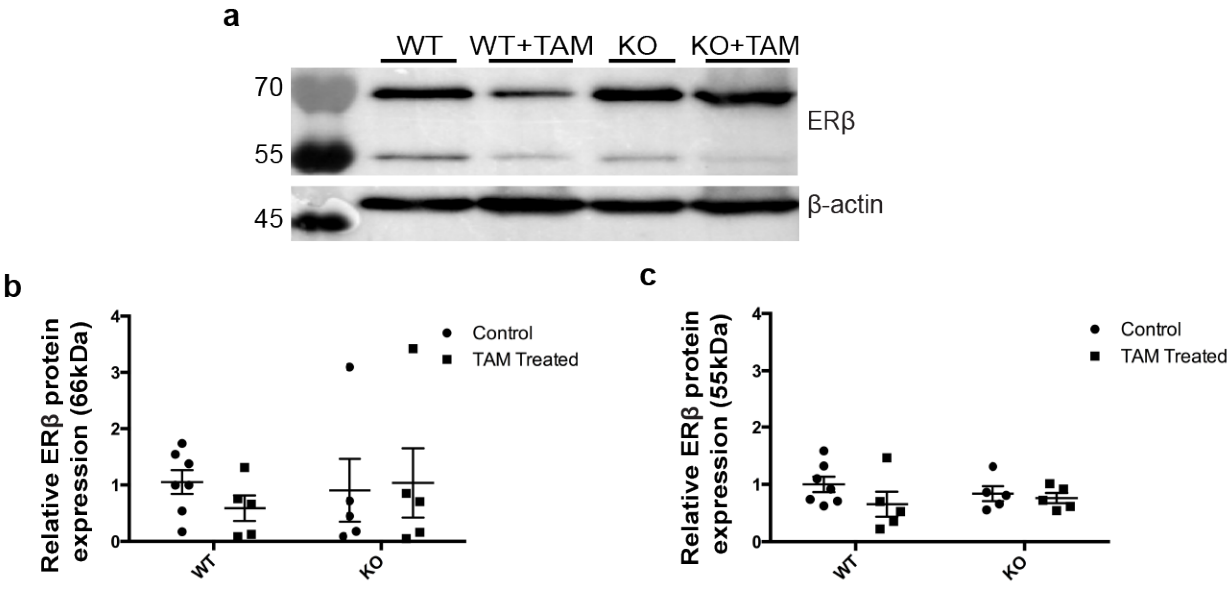

Supplementary Figure 4. Effect of tamoxifen on the levels of estrogen receptor beta.
a. Representative Western blot for ERβ*,* with β-actin and loading control for WT and *Mtm1* KO mice, and tamoxfen (TAM) treated WT and *Mtm1* KO mice; position of the molecular weight markers indicated (in kDa). All samples derive from the same experiment and blots were processed in parallel. Relative protein levels of the 66kDa isoform **b.** and 55kDa isoform **c.** of ER*β***,** determined by densitometry, standardized to β-actin and represented as the fold difference from the average of the WT (n=5 mice per group; graphs represent five technical replicates and mean ± SEM). Statistical comparisons were conducted by two-way ANOVA, followed by Tukey's multiple comparisons posttest. No statistical differences were observed.

a

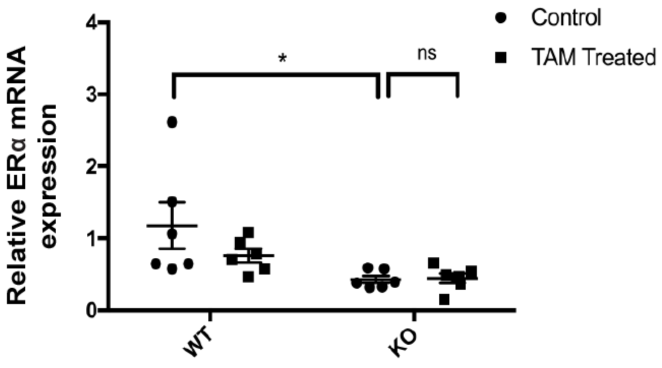

Supplementary Figure 5. Effect of Tamoxifen on estrogen receptor alpha transcript levels
a. Graph depicting mRNA levels of estrogen receptor alpha (ERα) in quadriceps of WT and *Mtm1* KO mice +/- tamoxifen (TAM) treatment. Expression level of ERα mRNA was determined by real-time qPCR and analyzed by the $2^{-\Delta\Delta CT}$ method. Values were normalized to TBP and represent the fold difference from the average of the WT (n=6 mice per group for ERα, graph represents 3 technical replicates for each mouse, 3 biological replicates and ± SEM). Of note, these results are consistent with the expression levels determined by RNA-seq. Statistical analysis was conducted using two-way ANOVA followed by Tukey's multiple comparisons post-test. *$P \leq 0.05$.

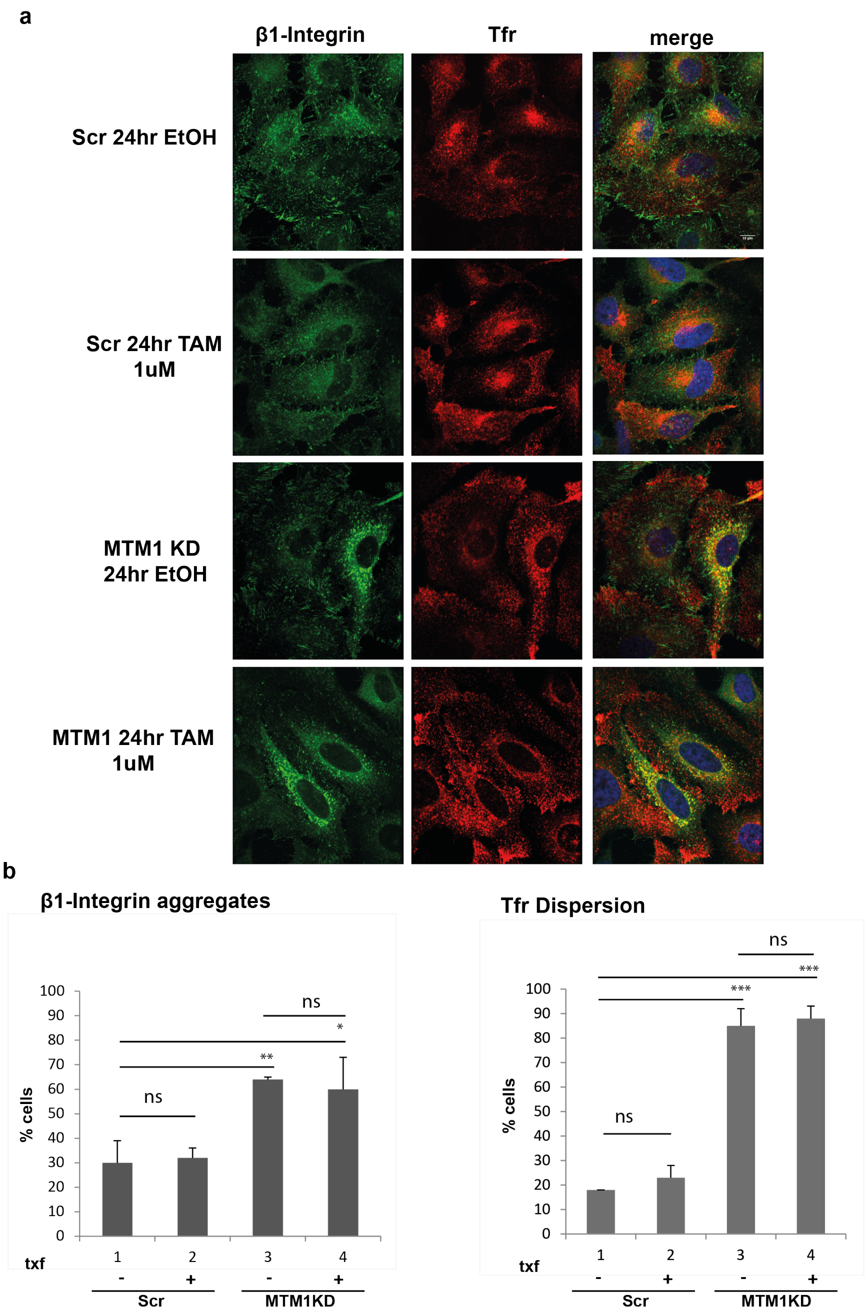

Supplementary Figure 6. Tamoxifen treatment (24hr) does not restore stalled endosomes in HeLa cells depleted of *MTM1*. a. Confocal images of β1-integrin and transferrin receptor (TfR) distribution in HeLa cells treated with scrambled (scr) siRNA or siRNA against *MTM1* and with 1 μM tamoxifen or ethanol (vehicle). **b.** Fraction of cells displaying β1-integrin aggregates or transferrin receptor dispersion (n=3). n = number of independent experiments with 11 images analyzed per condition per experiment. Mean ± SEM., *P <0.05, **P<0.01, P<0.001, one-way ANOVA. Representative images from one of the three independent experiments are shown in **a**. Scale bar = 10μM

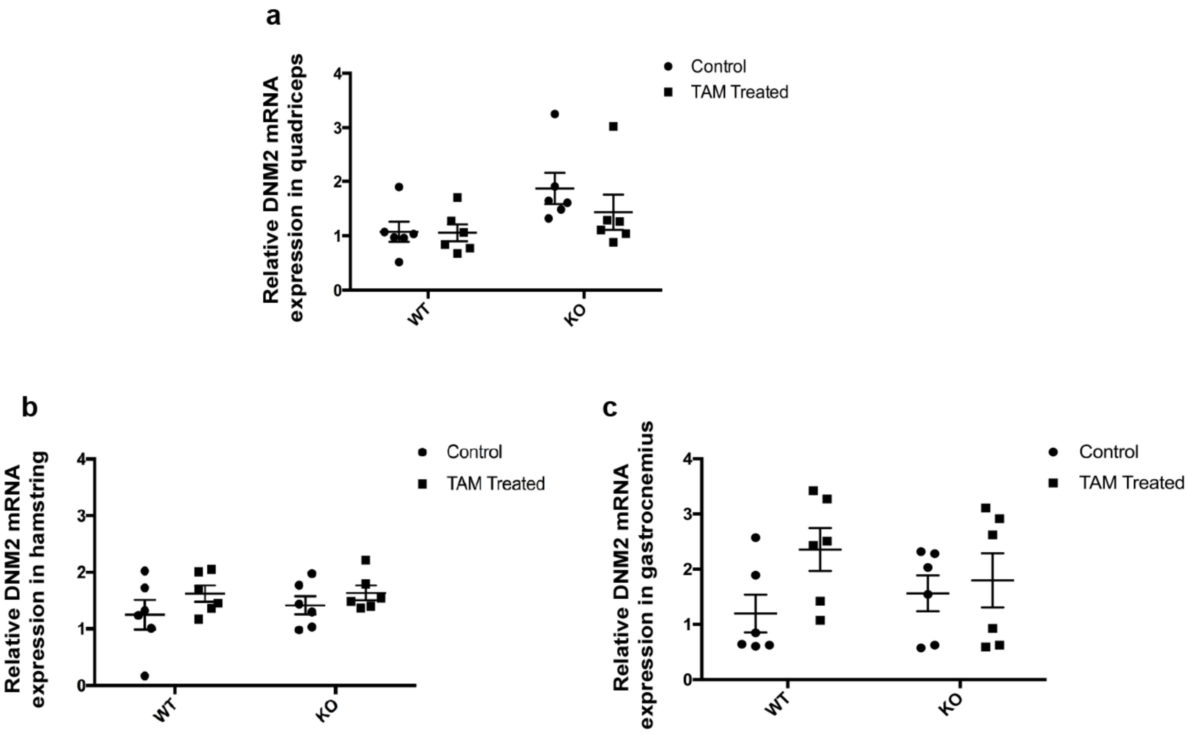

Supplementary Figure 7. Effect of Tamoxifen on dynamin-2 transcript levels.
Graphs depicting mRNA levels of dynamin-2 (DNM2) in **a.** quadriceps, **b.** hamstring and **c.** gastrocnemius muscle of WT and *Mtm1* KO mice +/- tamoxifen (TAM) treatment. Expression level of DNM2 mRNA was determined by real-time qPCR and analyzed by the $2^{-\Delta\Delta CT}$ method. Values were normalized to actin and represent the fold difference from the average of the WT (n=6 mice per group for DNM2, graph represents 3 technical replicates for each mouse, 3 biological replicates and ± SEM). Of note, DNM2 mRNA expression by qRT-PCR is consistent with the expression levels determined by RNA-seq. All statistical analyses were conducted using two-way ANOVA followed by Tukey's multiple comparisons post-test. No significant differences were observed.

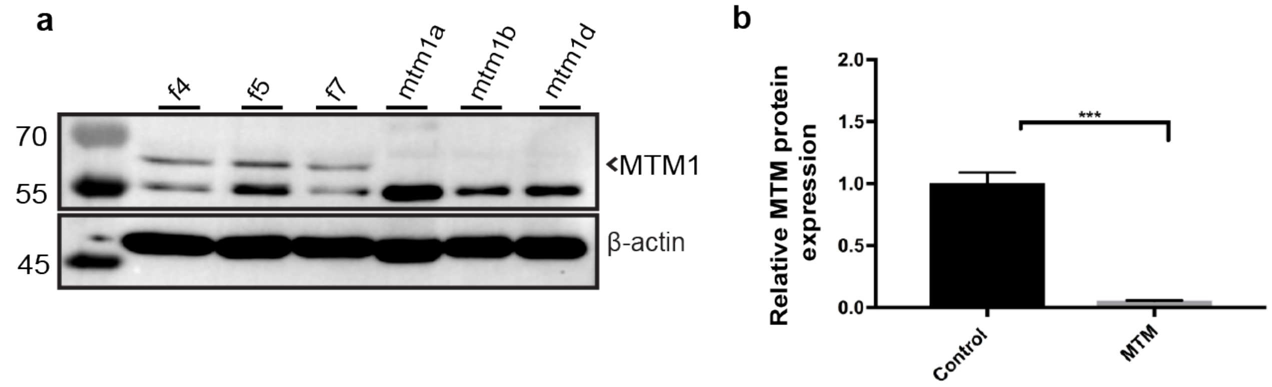

Supplementary Figure 8. MTM1 protein levels in primary human fibroblasts.

a. Representative Western blot for MTM1 with β-actin loading control in untreated human fibroblasts from controls (n=3) and MTM patients (n=3). Molecular weight indicated (in kDa). Arrow indicates the absence of the 60kDa MTM1 protein in lines mtm1a, mtm1b and mtm1d. All three samples from MTM patients have confirmed mutations in *MTM1*. **b.** Relative protein levels of MTM1 determined by densitometry, standardized to β-actin and represented as the fold difference from the average of the unaffected control (n=3 cell types per group; graph represents mean ± SEM). Statistical analyses were conducted by one-way ANOVA, followed by Tukey's multiple comparisons posttest. ***$P \leq 0.001$.

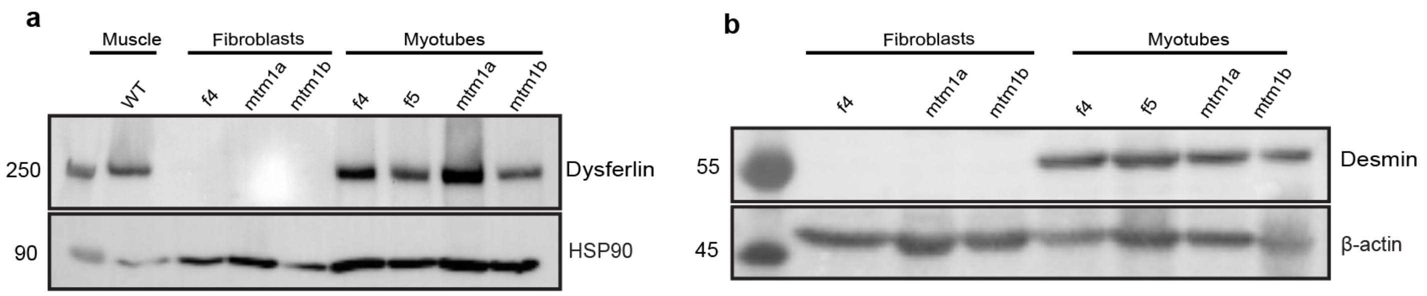

Supplementary Figure 9. Expression level of Dysferlin and Desmin in transdifferentiated human myotubes. a. Representative Western blot for Dysferlin with HSP90 loading control in WT murine muscle, untreated primary human fibroblasts and untreated transdifferentiated human myotubes (MTM = patient with myotubular myopathy). **b.** Representative Western blot for Desmin with β-actin loading control in untreated primary human fibroblasts and transdifferentiated myotubes (MTM = patient with myotubular myopathy). Molecular weight indicated (in kDa). Dysferlin and Desmin are both muscle-specific markers used here to indicate the successful transdifferentiation of control and MTM human fibroblasts into skeletal myotubes.

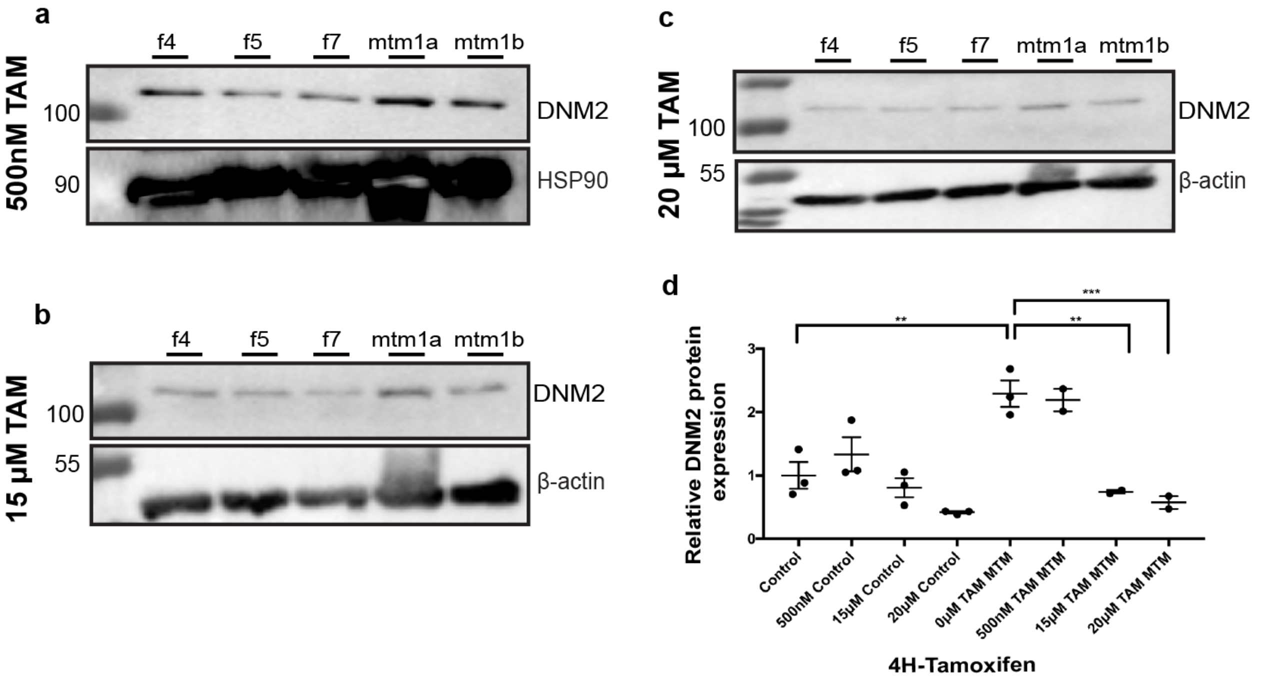

Supplementary Figure 10. Dose-dependent effect of *in-vitro* tamoxifen treatment on dynamin-2 protein levels in human fibroblasts. (a-c) Representative Western blots for DNM2, with β-actin and HSP90 loading controls in human fibroblasts treated with 500 nM, 15 μM and 20 μM tamoxifen; position of the molecular weight markers indicated (in kDa). All samples derive from the same experiment, with one to two separate blots run in parallel for each concentration (n= 3 biological replicates for controls, and 2 biological replicates for MTM). **d.** Relative protein levels of DNM2 in each treatment determined by densitometry, standardized to β-actin or HSP90 and represented as the fold difference from the average of the control (0μM TAM); graph represents 1 technical replicate for 500nM and 2 technical replicates 15 μM and 20 μM TAM, and mean ± SEM). Statistical comparisons were made by one-way ANOVA followed by Tukey's multiple comparisons posttest. $^{**}P \leq 0.01$, $^{***}P \leq 0.001$.

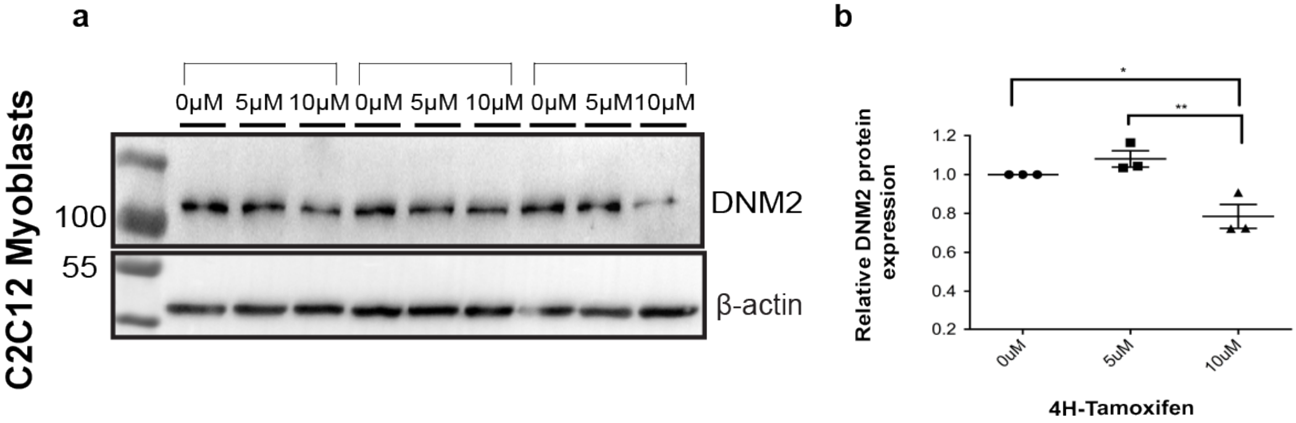

Supplementary Figure 11. Dose-dependent effects of *in vitro* tamoxifen treatment on C2C12 Myoblasts. a. Representative Western blot for DNM2 in C2C12 myoblasts treated with 5μM and 10 μM of tamoxifen, with β-actin loading control; molecular weight indicated (in kDa). All samples derive from the same experiment and the blot was run with each sample in triplicate. **b.** Relative protein levels of DNM2 determined by densitometry, standardized to β-actin and represented as the fold difference from the average of the untreated control (0μM TAM) (n=3; graph represents three technical replicates and mean ± SEM). Dose dependent effect of 10μM tamoxifen reduced DNM2 levels to 0.785 +/- 0.061 (P= 0.0283) compared to 0μM TAM controls. Statistical analyses were conducted by one-way ANOVA, followed by Tukey's multiple comparisons posttest. *$P \leq 0.05$, **$P \leq 0.01$.

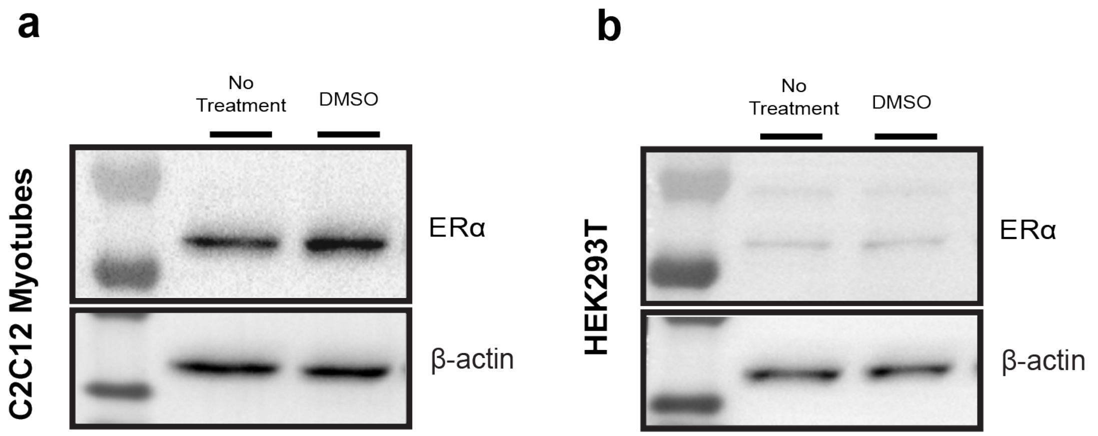

Supplementary Figure 12. Expression level of estrogen receptor alpha in C2C12 myotubes and HEK293T cells. (a-b) Representative Western blot for ERα with β-actin loading controls in untreated C2C12 myotubes and HEK293T cells. Molecular weight indicated (in kDa). The 55kDa isoform of ERα is highly expressed in C2C12 cells, whereas both the 66kDa and 55kDa isoform of ERα are negligibly expressed in HEK293T cells.

Uncropped Blots _ Tamoxifen Therapy in a Murine Model of Myotubular Myopathy

Figure 6
a. ERa + actin

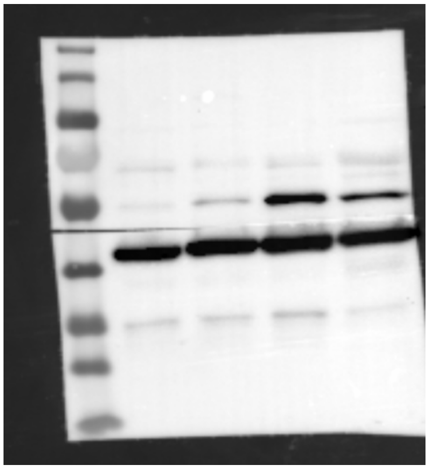

Figure 6
b. ERa + actin

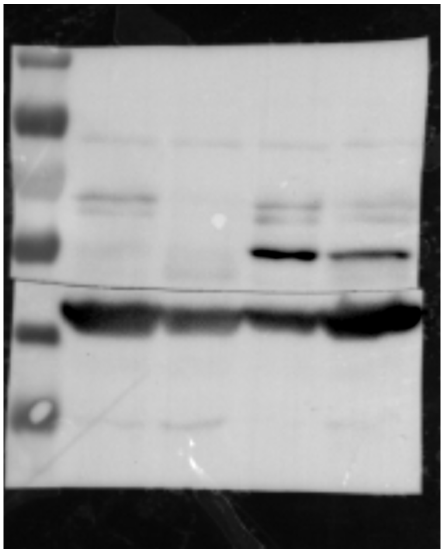

Figure 6
c. ERa + actin

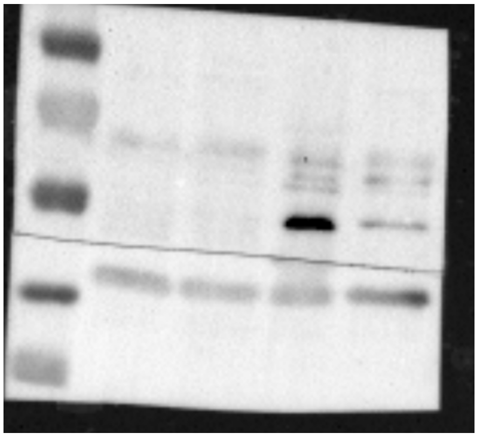

Figure 7
a. PIK3C2B

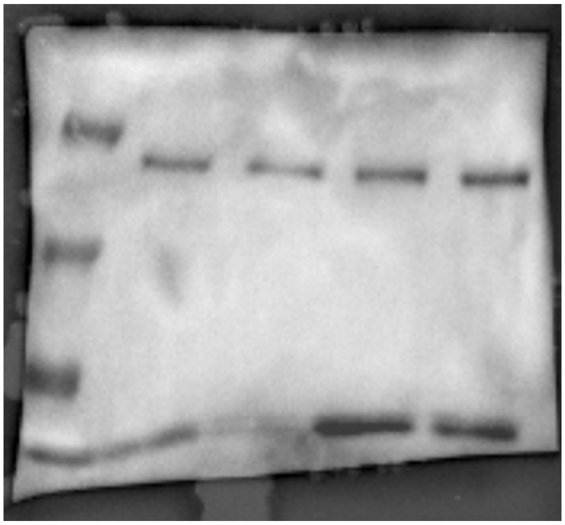

Figure 7
a. HSP90

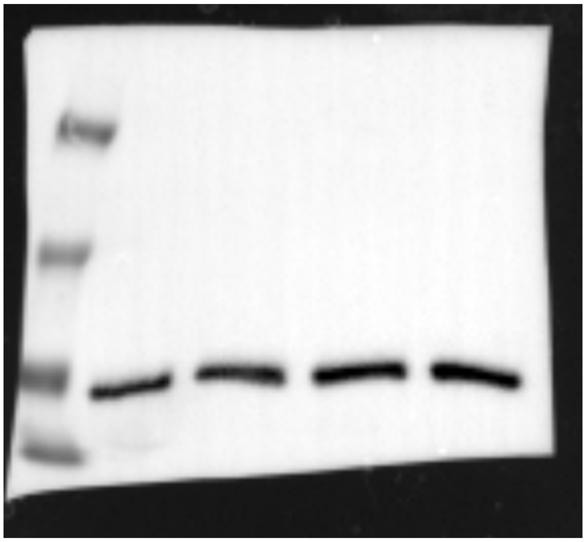

Figure 7
b. PIK3C2B

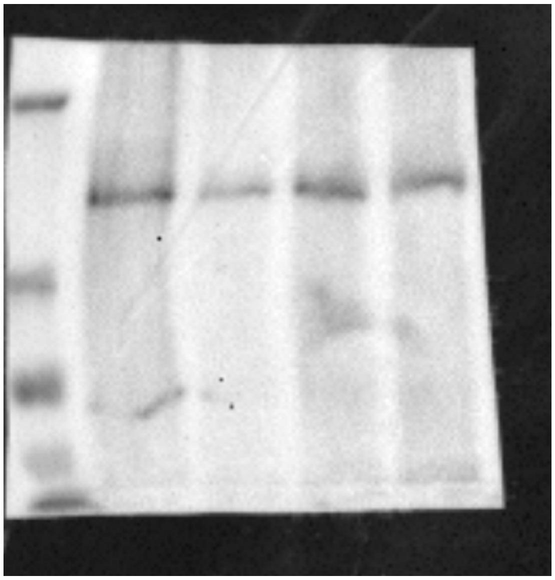

Figure 7
b. HSP90

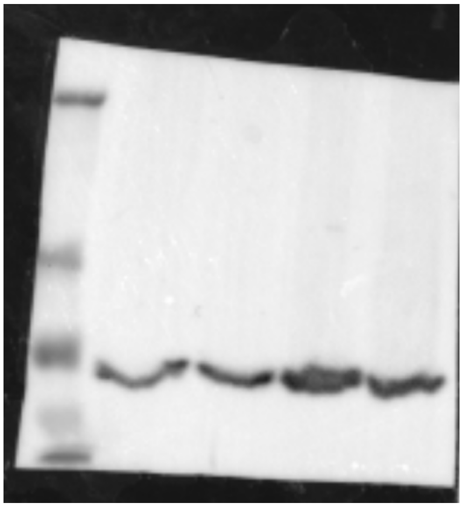

Figure 7
c. PIK3C2B

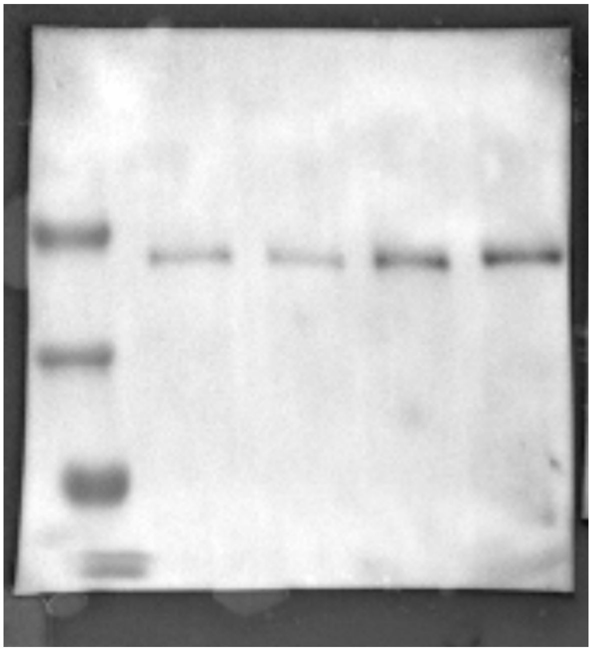

Figure 7
c. HSP90

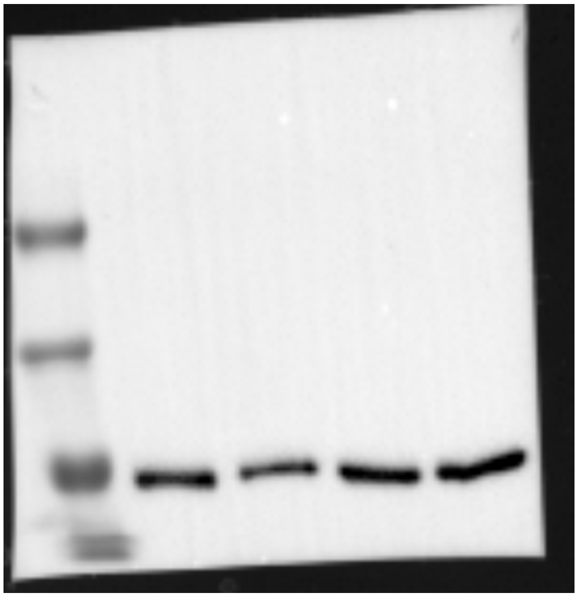

Figure 8
a. DNM2

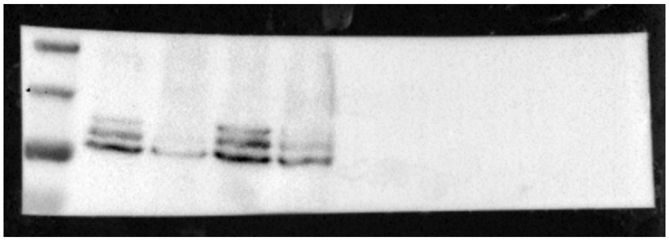

Actin

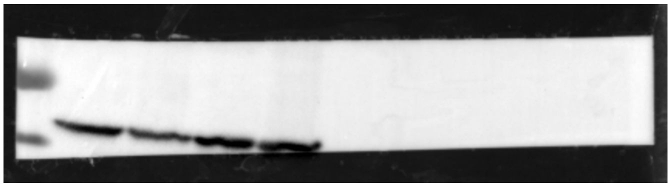

Figure 8
b. DNM2 + actin

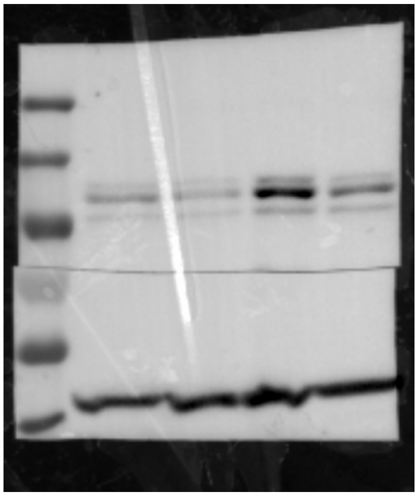

Figure 8
c. DNM2 + actin

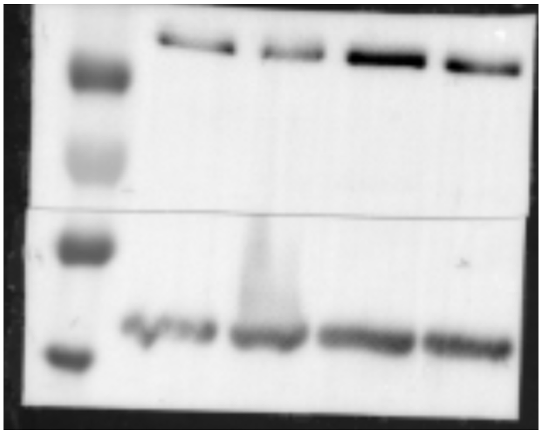

Figure 8
d. DNM2

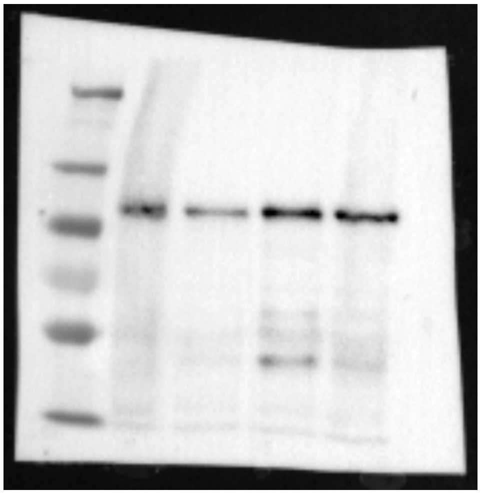

Figure 8
d. Actin

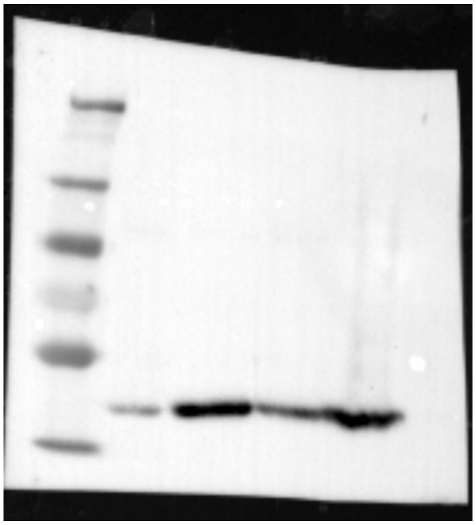

Figure 8
m. DNM2

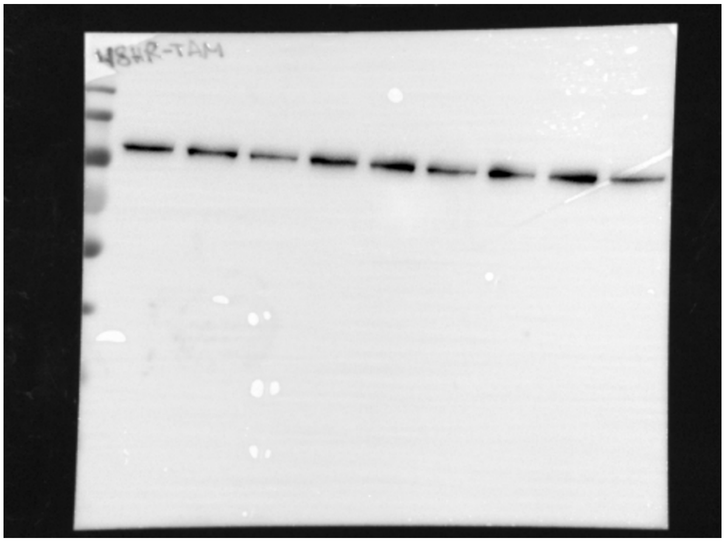

Figure 8
m. Actin

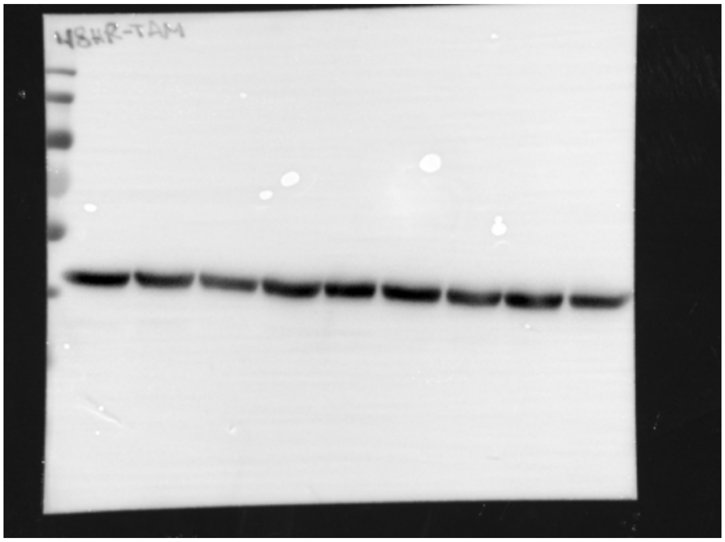

Figure 8
n. DNM2 + actin

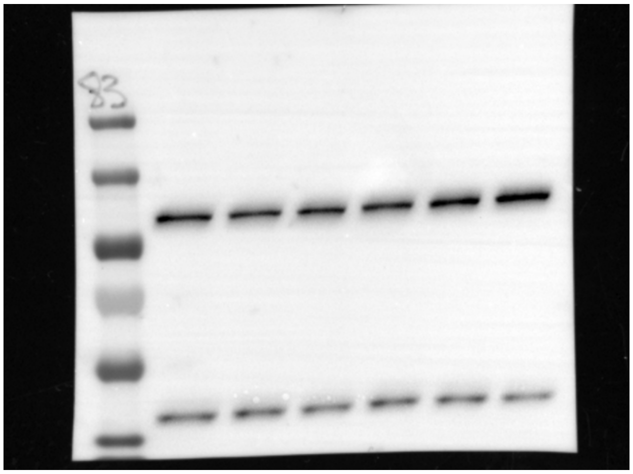

Figure 8
n. Actin

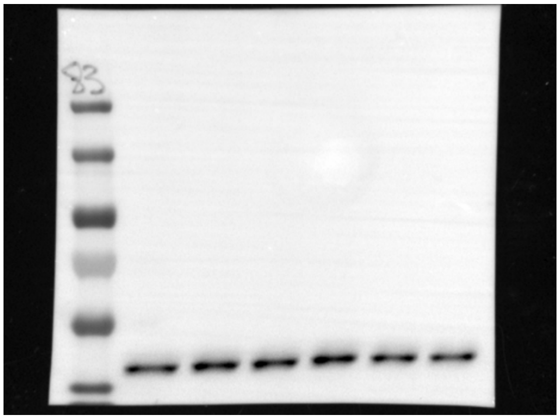

Figure 8
i. 0uM TAM
DNM2

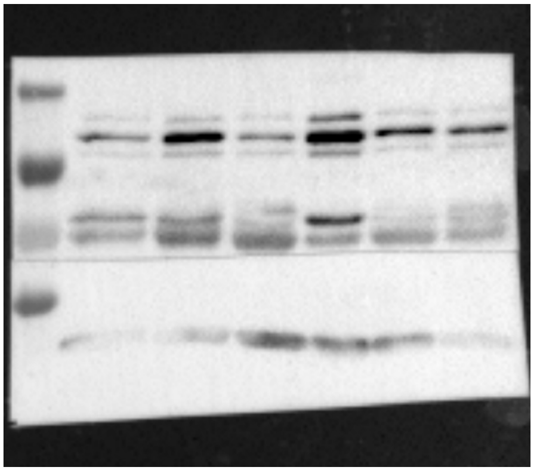

Figure 8
i. 0uM TAM
Actin

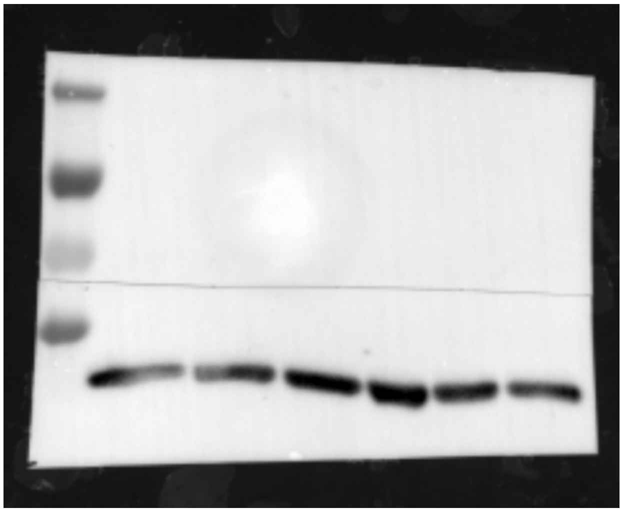

Figure 8
i. 10uM TAM
DNM2 + actin

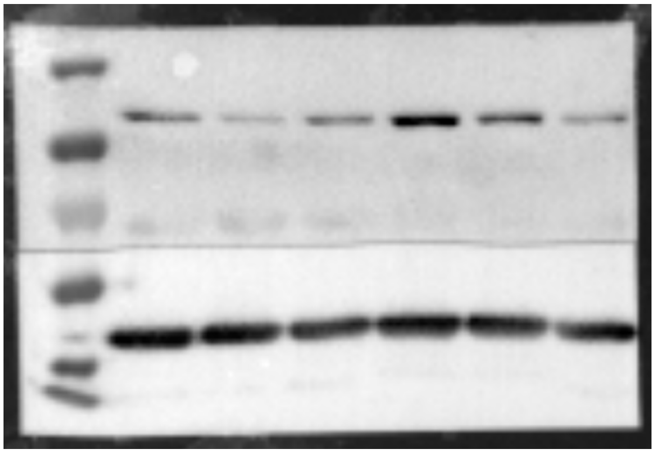

Figure 8
j. 0uM TAM
DNM2 + actin

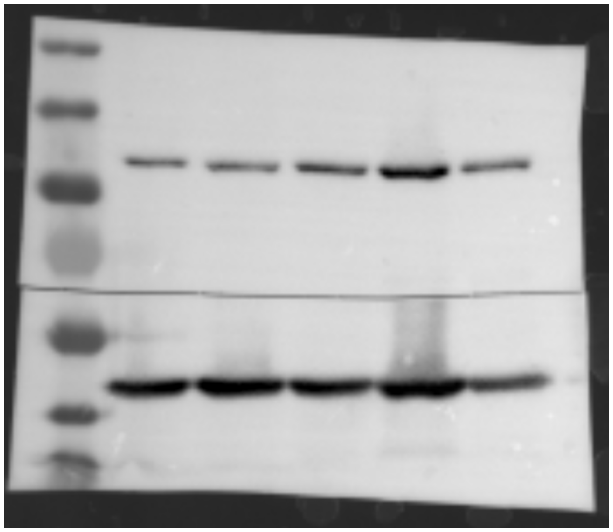

Figure 8
j. 10uM TAM
DNM2 + actin

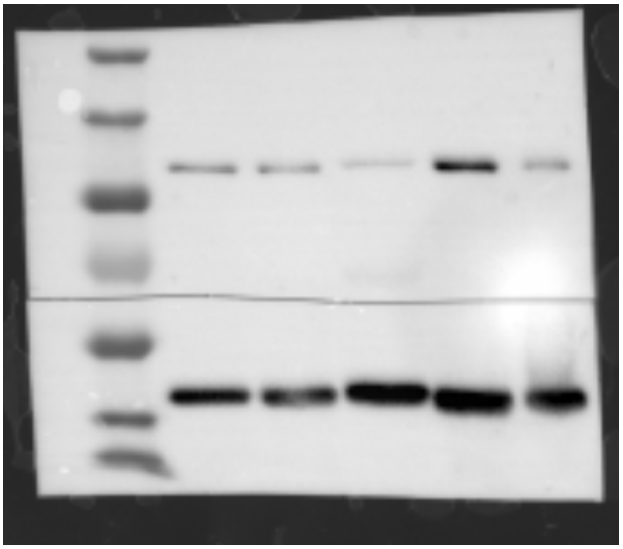

Figure 8
q. DNM2 + Actin

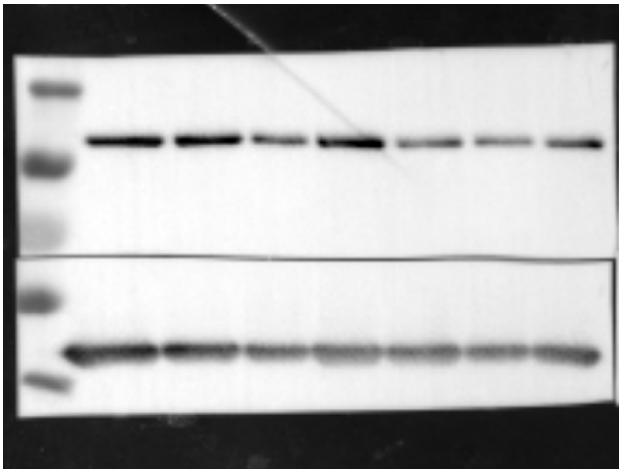

Figure 8
r. DNM2

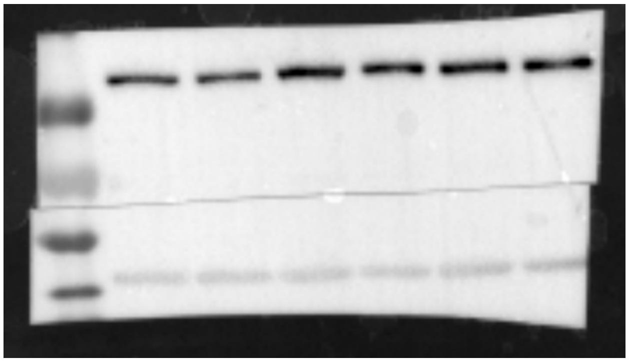

Figure 8
r. Actin

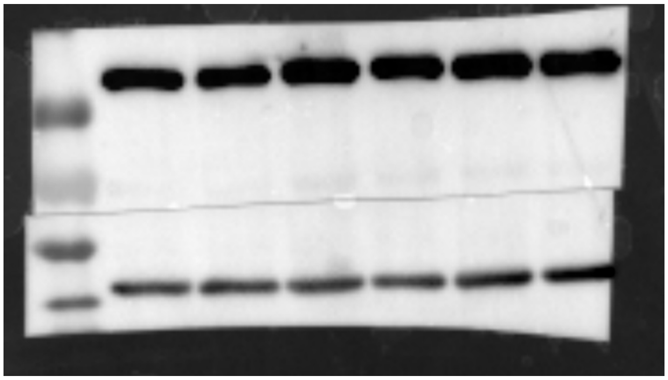

Supplementary Figure 4
a. ERb + actin

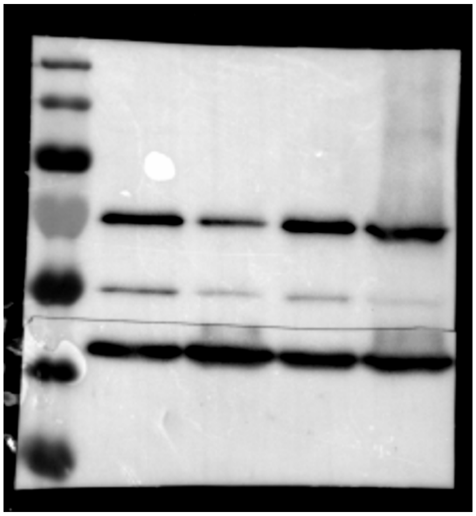

Supplementary Figure 8.
a. MTM1 + actin

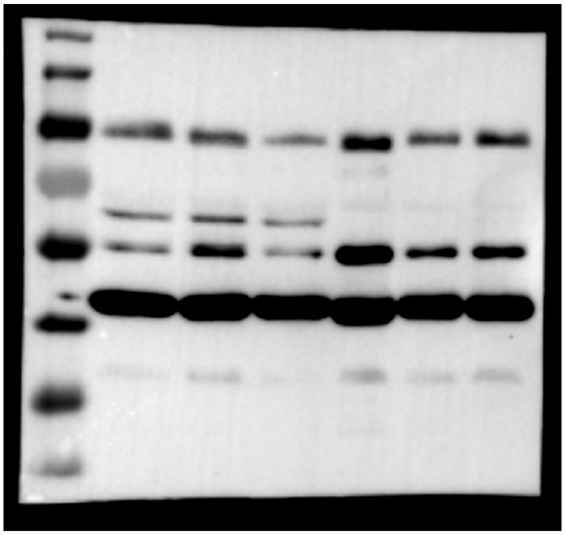

Supplementary Figure 9.
a. Dysferlin

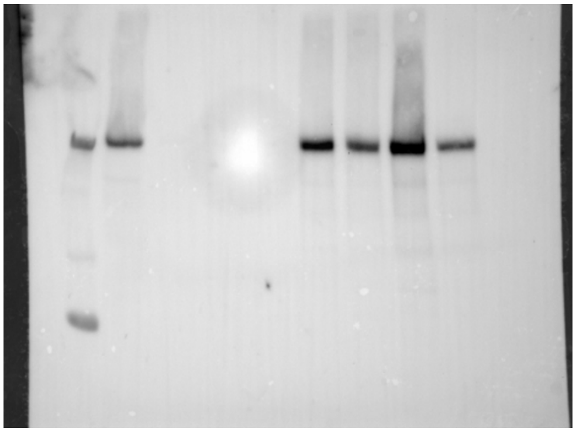

Supplementary Figure 9.
a. HSP90

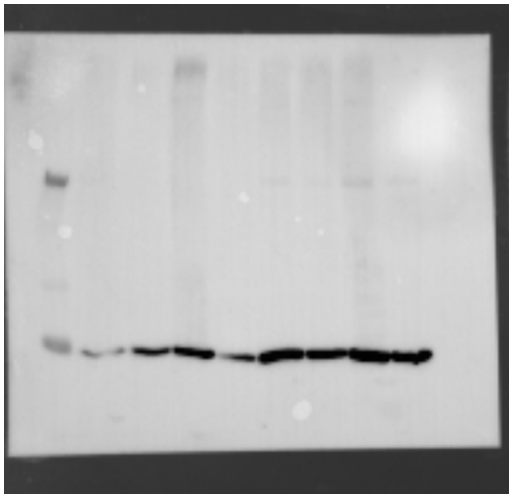

Supplementary Figure 9.
b. Desmin

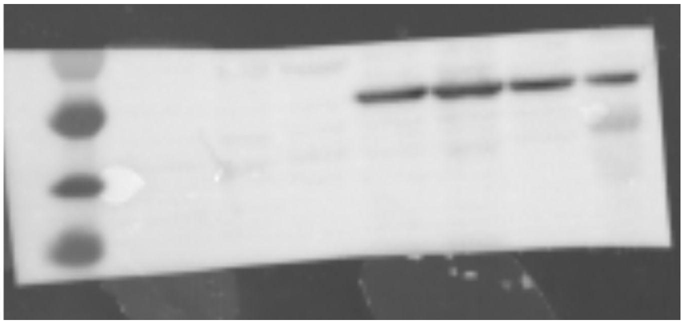

Supplementary Figure 9.
b. Actin

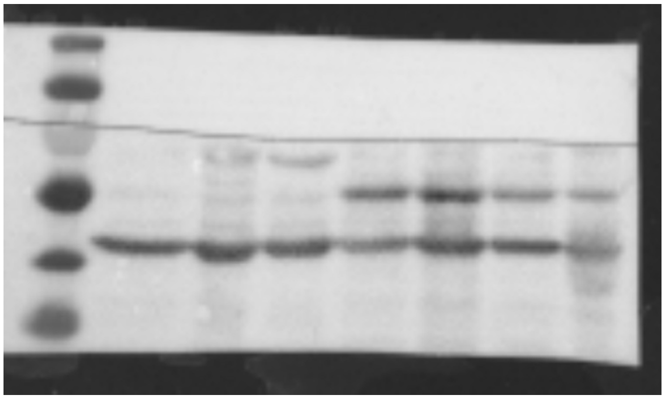

Supplementary Figure 10.
a. DNM2

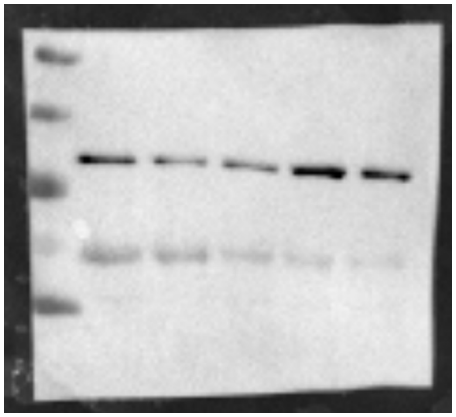

Supplementary Figure 10.
a. HSP90

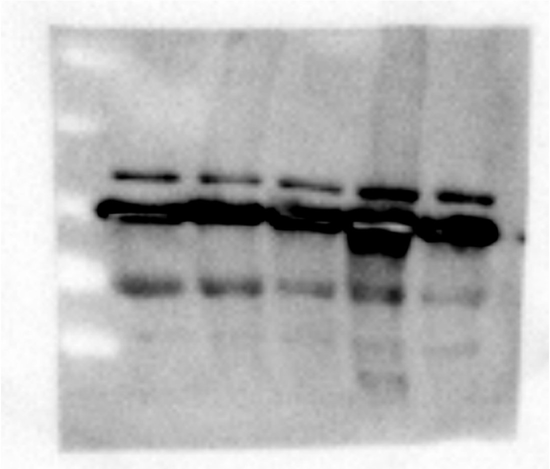

Supplementary Figure 10.
b. DNM2 + actin

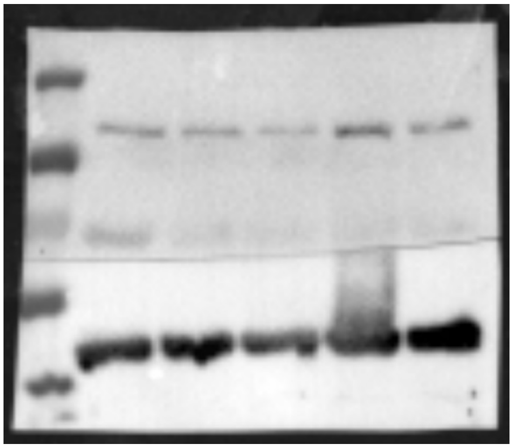

Supplementary Figure 10.
c. DNM2 + actin

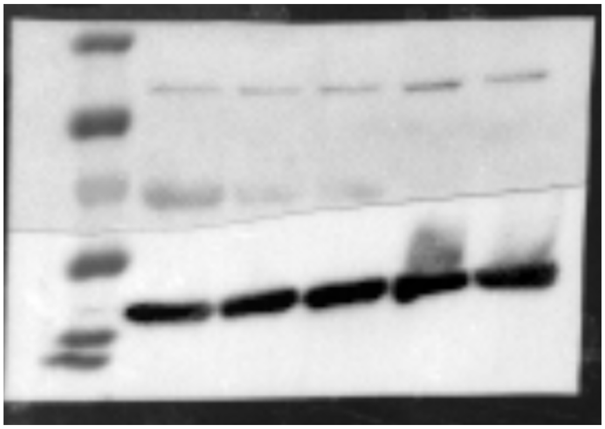

Supplementary Figure 11.
a. DNM2

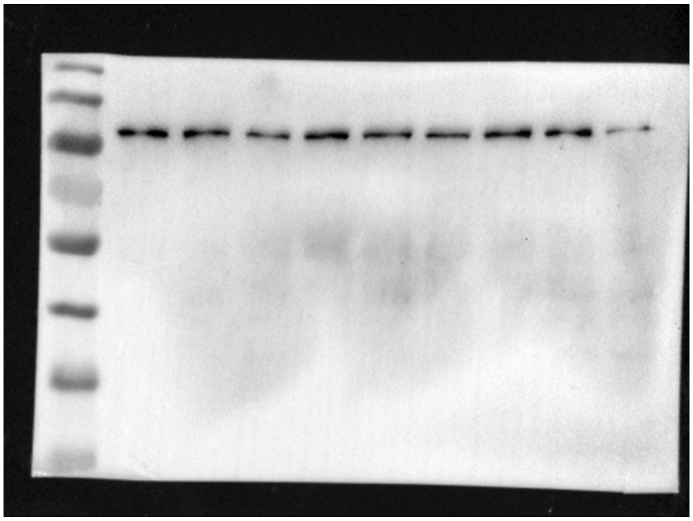

Supplementary Figure 11.
a. Actin

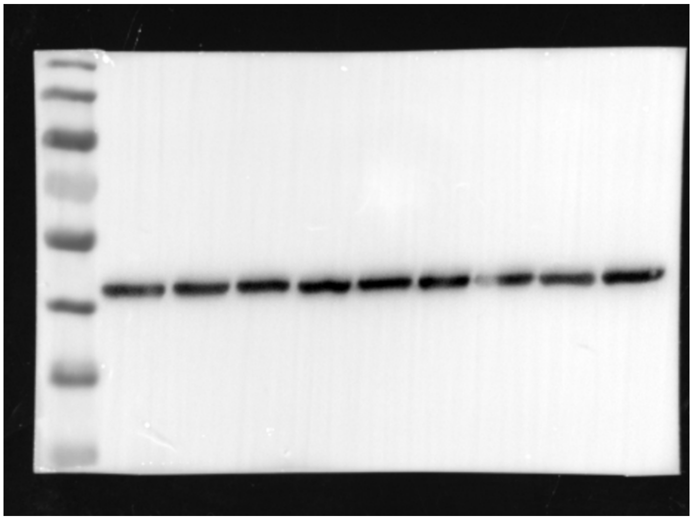

Supplementary Figure 12.
a. ERa

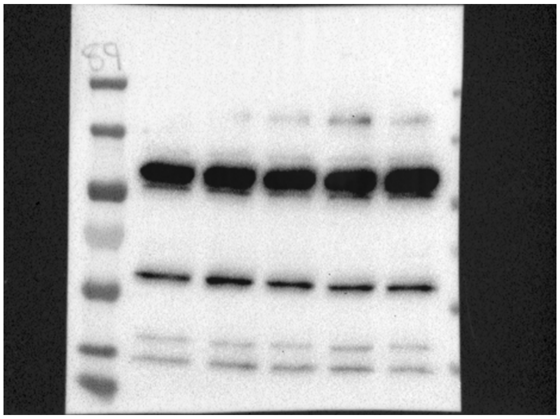

Supplementary Figure 12.
a. Actin

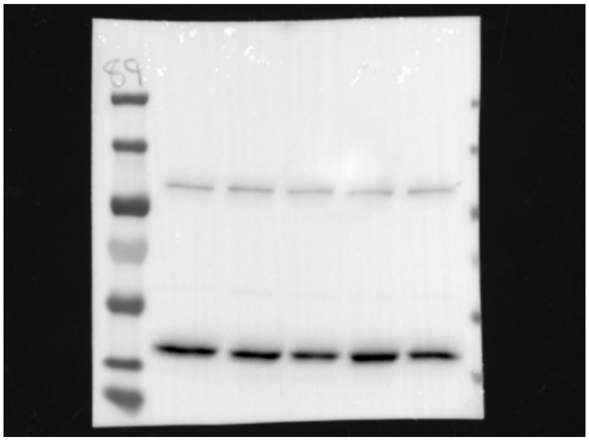

Supplementary Figure 12.
b. ERa

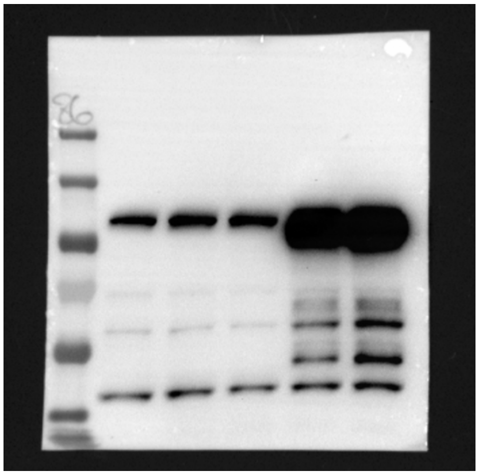

Supplementary Figure 12.

b. Actin

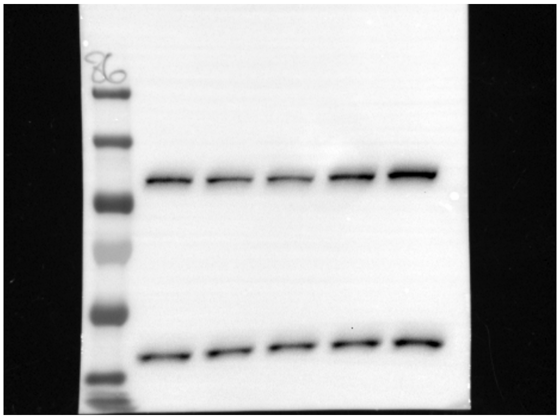

Supplement: Supplementary file 1 — Supplementary Information [file 41467_2018_7057_MOESM1_ESM.pdf]
