# Supplementary material for: Tamoxifen therapy in a murine model of myotubular myopathy
Source: Nat Commun. 2018 Nov 19;9:4849. doi: 10.1038/s41467-018-07057-5 (PMC6242823; doi:10.1038/s41467-018-07057-5)
Supplement: Supplementary file 12 — Description of Additional Supplementary Files [file 41467_2018_7057_MOESM12_ESM.pdf]

Supplementary Movie 1: High dose tamoxifen started at 21 days improves *Mtm1* KO motor function.

Video of 29 day old wild type and *Mtm1* KO mice treated with high dose tamoxifen (TAM) and of an untreated *Mtm1* KO littermate. Note that the TAM treated *Mtm1* KO moves normally around the cage and can climb the sides of the container, while the untreated *Mtm1* KO has very limited movement.

Su**pplementary Movie 2: High dose tamoxifen started at 21 days extends survival and preserves motor function of *Mtm1* KO mice.**

Video of 69 day old wild type and *Mtm1* KO mice treated with tamoxifen starting at 21 days of age. While the *Mtm1* KO does not move as well as the WT, it can still move freely around the cage. No untreated *Mtm1* KO mice survive to this age.

Supplementary Movie 3: Low dose tamoxifen started at 21 days improves motor function of Mtm1 KO mice.

Video of 29 day old wild type and *Mtm1* KO mice treated with low dose tamoxifen and of an untreated *Mtm1* KO littermate. As compared to the untreated mouse, the TAM treated *Mtm1* KO moves more easily and quickly around the cage.

Supplementary Movie 4: Low dose tamoxifen started at 21 days extends survival and improves motor function of *Mtm1* KO mice.

Video of a 68 day old wild type *Mtm1* KO mouse, and a wild type littermate, treated with low dose tamoxifen. The *Mtm1* KO mouse is still able to move, though has clear weakness as compared to the WT littermate. No untreated *Mtm1* KOs survive to this age.

Supplemental Movie 5: Low dose tamoxifen started at 14 days ameliorates the phenotype *Mtm1* KO mice.

Video of 28 day old mice treated with tamoxifen starting at 14 days of age. The *Mtm1* KOs appear indistinguishable from their wild type littermates.

Supplementary Movie 6: Estradiol improves motor function and extends survival of *Mtm1* KO mice

Video of a 40 day old *Mtm1* KO mouse treated with estradiol, as well as an *Mtm1* KO treated with tamoxifen and a KO without treatment. Both treated KO mice move freely around the cage and appear relatively indistinguishable from each other. The untreated KO has very limited movement and obvious hindlimb weakness.

Supplemental Movie 7: Fulvestrant does not improve the *Mtm1* KO phenotype

Video of a 28 day old Mtm1 KO mouse treated with fulvestrant, and Mtm1 KO littermate that received only the carrier solvent (miglyol). Fulvestrant does not improve motor function in the KO mice.

Supplementary Dataset 1: Spreadsheet of transcripts significantly enriched with comparative RNA sequencing.

RNA sequencing was performed on skeletal muscle extracts from wild type (WT) and Mtm1 knockout (KO) mice +/- tamoxifen (TAM) treatment. Studies were performed at 35 days of age on samples from littermates, with treatment or placebo started at 21 days of age. The excel sheet presents all significantly changed transcripts in the following comparisons: WT vs KO, WT vs WT+TAM, KO vs KO+TAM, and WT+TAM vs KO+TAM.

Supplementary Dataset 2 : Summary of GO terms from comparative RNA sequencing.

GO terms cluster analysis was performed from the RNA sequencing data. Comparisons are made between WT vs. KO, WT vs. WT+TAM, WT+TAM vs. KO+TAM. Of note, there were no GO terms significantly enriched in the comparison between WT and WT+TAM.
